# CaSTH2 disables CaWRKY40 from activating pepper thermotolerance and immunity against *Ralstonia solanacearum* via physical interaction

**DOI:** 10.1093/hr/uhae066

**Published:** 2024-03-02

**Authors:** Xingge Cheng, Meiyun Wan, Yuqiu Song, Qian Liu, Xiaohui Hu, Xiufang Chen, Xujing Zhang, Yapeng Zhang, Ruijie Wu, Qiaoling Lu, Yu Huang, Jingang Lv, WeiWei Cai, Deyi Guan, Sheng Yang, Shuilin He

**Affiliations:** National Education Ministry Key Laboratory of Plant Genetic Improvement and Comprehensive Utilization, Fujian Agriculture and Forestry University, Fuzhou, Fujian 350002, China; Key Laboratory of Applied Genetics of Universities in Fujian Province, Fujian Agriculture and Forestry University, Fuzhou, Fujian 350002, China; College of Agriculture, Fujian Agriculture and Forestry University, Fuzhou, Fujian 350002, China; National Education Ministry Key Laboratory of Plant Genetic Improvement and Comprehensive Utilization, Fujian Agriculture and Forestry University, Fuzhou, Fujian 350002, China; Key Laboratory of Applied Genetics of Universities in Fujian Province, Fujian Agriculture and Forestry University, Fuzhou, Fujian 350002, China; College of Agriculture, Fujian Agriculture and Forestry University, Fuzhou, Fujian 350002, China; National Education Ministry Key Laboratory of Plant Genetic Improvement and Comprehensive Utilization, Fujian Agriculture and Forestry University, Fuzhou, Fujian 350002, China; Key Laboratory of Applied Genetics of Universities in Fujian Province, Fujian Agriculture and Forestry University, Fuzhou, Fujian 350002, China; College of Agriculture, Fujian Agriculture and Forestry University, Fuzhou, Fujian 350002, China; National Education Ministry Key Laboratory of Plant Genetic Improvement and Comprehensive Utilization, Fujian Agriculture and Forestry University, Fuzhou, Fujian 350002, China; Key Laboratory of Applied Genetics of Universities in Fujian Province, Fujian Agriculture and Forestry University, Fuzhou, Fujian 350002, China; College of Life Sciences, Fujian Agriculture and Forestry University, Fuzhou, Fujian 350002, China; National Education Ministry Key Laboratory of Plant Genetic Improvement and Comprehensive Utilization, Fujian Agriculture and Forestry University, Fuzhou, Fujian 350002, China; Key Laboratory of Applied Genetics of Universities in Fujian Province, Fujian Agriculture and Forestry University, Fuzhou, Fujian 350002, China; College of Agriculture, Fujian Agriculture and Forestry University, Fuzhou, Fujian 350002, China; National Education Ministry Key Laboratory of Plant Genetic Improvement and Comprehensive Utilization, Fujian Agriculture and Forestry University, Fuzhou, Fujian 350002, China; Key Laboratory of Applied Genetics of Universities in Fujian Province, Fujian Agriculture and Forestry University, Fuzhou, Fujian 350002, China; College of Agriculture, Fujian Agriculture and Forestry University, Fuzhou, Fujian 350002, China; National Education Ministry Key Laboratory of Plant Genetic Improvement and Comprehensive Utilization, Fujian Agriculture and Forestry University, Fuzhou, Fujian 350002, China; Key Laboratory of Applied Genetics of Universities in Fujian Province, Fujian Agriculture and Forestry University, Fuzhou, Fujian 350002, China; College of Life Sciences, Fujian Agriculture and Forestry University, Fuzhou, Fujian 350002, China; National Education Ministry Key Laboratory of Plant Genetic Improvement and Comprehensive Utilization, Fujian Agriculture and Forestry University, Fuzhou, Fujian 350002, China; Key Laboratory of Applied Genetics of Universities in Fujian Province, Fujian Agriculture and Forestry University, Fuzhou, Fujian 350002, China; College of Agriculture, Fujian Agriculture and Forestry University, Fuzhou, Fujian 350002, China; National Education Ministry Key Laboratory of Plant Genetic Improvement and Comprehensive Utilization, Fujian Agriculture and Forestry University, Fuzhou, Fujian 350002, China; Key Laboratory of Applied Genetics of Universities in Fujian Province, Fujian Agriculture and Forestry University, Fuzhou, Fujian 350002, China; College of Agriculture, Fujian Agriculture and Forestry University, Fuzhou, Fujian 350002, China; National Education Ministry Key Laboratory of Plant Genetic Improvement and Comprehensive Utilization, Fujian Agriculture and Forestry University, Fuzhou, Fujian 350002, China; Key Laboratory of Applied Genetics of Universities in Fujian Province, Fujian Agriculture and Forestry University, Fuzhou, Fujian 350002, China; College of Agriculture, Fujian Agriculture and Forestry University, Fuzhou, Fujian 350002, China; National Education Ministry Key Laboratory of Plant Genetic Improvement and Comprehensive Utilization, Fujian Agriculture and Forestry University, Fuzhou, Fujian 350002, China; Key Laboratory of Applied Genetics of Universities in Fujian Province, Fujian Agriculture and Forestry University, Fuzhou, Fujian 350002, China; College of Agriculture, Fujian Agriculture and Forestry University, Fuzhou, Fujian 350002, China; National Education Ministry Key Laboratory of Plant Genetic Improvement and Comprehensive Utilization, Fujian Agriculture and Forestry University, Fuzhou, Fujian 350002, China; Key Laboratory of Applied Genetics of Universities in Fujian Province, Fujian Agriculture and Forestry University, Fuzhou, Fujian 350002, China; College of Agriculture, Fujian Agriculture and Forestry University, Fuzhou, Fujian 350002, China; College of of Horticultural Sciences, Zhejiang Agriculture and Forestry University, Hangzhou, Zhejiang, 350002, China; National Education Ministry Key Laboratory of Plant Genetic Improvement and Comprehensive Utilization, Fujian Agriculture and Forestry University, Fuzhou, Fujian 350002, China; Key Laboratory of Applied Genetics of Universities in Fujian Province, Fujian Agriculture and Forestry University, Fuzhou, Fujian 350002, China; College of Agriculture, Fujian Agriculture and Forestry University, Fuzhou, Fujian 350002, China; National Education Ministry Key Laboratory of Plant Genetic Improvement and Comprehensive Utilization, Fujian Agriculture and Forestry University, Fuzhou, Fujian 350002, China; Beijing Key Laboratory of Growth and Developmental Regulation for Protected Vegetable Crops, Department of Vegetable Science, College of Horticulture, China Agricultural University, Beijing, 100193, China; National Education Ministry Key Laboratory of Plant Genetic Improvement and Comprehensive Utilization, Fujian Agriculture and Forestry University, Fuzhou, Fujian 350002, China; Key Laboratory of Applied Genetics of Universities in Fujian Province, Fujian Agriculture and Forestry University, Fuzhou, Fujian 350002, China; College of Agriculture, Fujian Agriculture and Forestry University, Fuzhou, Fujian 350002, China

## Abstract

CaWRKY40 coordinately activates pepper immunity against *Ralstonia solanacearum* infection (RSI) and high temperature stress (HTS), forms positive feedback loops with other positive regulators and is promoted by CaWRKY27b/CaWRKY28 through physical interactions; however, whether and how it is regulated by negative regulators to function appropriately remain unclear. Herein, we provide evidence that CaWRKY40 is repressed by a SALT TOLERANCE HOMOLOG2 in pepper (CaSTH2). Our data from gene silencing and transient overexpression in pepper and epoptic overexpression in *Nicotiana benthamiana* plants showed that CaSTH2 acted as negative regulator in immunity against RSI and thermotolerance. Our data from BiFC, CoIP, pull down, and MST indicate that CaSTH2 interacted with CaWRKY40, by which CaWRKY40 was prevented from activating immunity or thermotolerance-related genes. It was also found that CaSTH2 repressed CaWRKY40 at least partially through blocking interaction of CaWRKY40 with CaWRKY27b/CaWRKY28, but not through directly repressing binding of CaWRKY40 to its target genes. The results of study provide new insight into the mechanisms underlying the coordination of pepper immunity and thermotolerance.

## Introduction

Under the selection pressure from various stresses in their nature habitats, plants have evolved defense mechanisms to defend themselves. They generally turn on defense responses by initiating defense signaling, amplifying and transmitting the signaling into the nucleus to activate massive transcriptional reprogramming, thereby activating appropriate defense reactions. Pathogen attack and high temperature stress (HTS) aretwo important stresses, which occur successively or simultaneously and are frequently encountered by plants in warm seasons in tropical and subtropical regions. These two stresses are distinct in their nature and differently perceived by plants: pathogens are recognized by plants with pathogen-associated molecular pattern (PAMP)-recognition receptors (PRRs) and intracellular R proteins [1–3], and are restrained by antimicrobial compounds, pathogenesis-related proteins or hypersensitive response (HR) [4–8], while HTS is sensed by sensors different from receptors that are used by plants to perceive pathogen infection [9–12], thereby initiating HTS responding signaling and activating plant thermotolerance by enhancing the synthesis of antioxidants, heat shock proteins (HSPs), and scavengers of reactive oxygen species (ROS) [13–15]. Despite these differences, plant immunity and thermotolerance share signaling elements such as Ca^2 + ^ [16], ROS [16], hormones including salicyclic acid (SA), jasmonate (JA) and abscisic acid (ABA) [17, 18], MAPK cascades [19], and transcription factors (TFs) [20–24], suggesting a role for these common components in the coordination of the immunity and thermotolerance of the plant, but the underlying mechanisms currently remain poorly understood.

Given that plant defense responses to both pathogen attack and HTS are mostly regulated at transcriptional level by a number of TFs [25–29], and some TFs are shared by plant response to pathogen attack and to HTS [20–24], these shared TFs might be related to the coordination of plant immunity and thermotolerance. WRKY proteins, which are distinguished by their unique binding to the W- (TTGACC/T) or WT-box and their highly conserved WRKY domain, constitute one of the largest plant transcription factor families [30, 31]. A growing body of research shows that many members of the WRKY family, found in a variety of plant species, including rice and Arabidopsis, have been linked to the control of plant immunity by interacting with hundreds or even thousands of genes related to immunity [26, 27, 32–34]; some of these WRKY proteins might be targeted by pathogens for their successful infection [35, 36]. WRKY proteins are also involved in plant thermotolerance [20, 24, 37–40], and some of them are shared by plant immunity and thermotolerance [20, 21]. To activate defense response fast and efficiently, these WRKYs operate by forming transcriptional cascades or networks [32], with some WRKY factors that hold central positions being post-translationally regulated by other proteins including WRKY, VQ protein, MAPK, chromatin remodeling proteins, calmodulin, 14-3-3, through protein–protein interaction [41–44]. Deconstructing these networks and revealing the mechanism of action of the key proteins are effective approaches to elucidate the molecular mechanisms underlying plant disease resistance.

B-box-containing proteins are characterized their N-terminal tandem repeated B-boxes, a Zn2 + −ligating domain that has been suggested to be a domain for protein interaction, consisting of conserved Cys and His residues, and there are 32 B-box-containing proteins known to exist in Arabidopsis [45]. SALT TOLERANCE HOMOLOG2 (STH2) has been found to act as activator of HY5 in concert with STH3 to promote its transcription via G-box in regulation of photomorphogenic development [45–47], while B-box zinc finger proteins such as B-box (BBX19), BBX21, or B-BBX32 is involved in photomorphogenesis regulation [48, 49], seed germination [50], or shade avoidance [51]. In addition, STH2 has been proposed to be a PR gene as it has been frequently found to be transcriptionally modified by pathogen infection in plants such as tomato and pepper [52–55], whose expression was reduced by the knockout of SlMYC2 [54], or upregulated by SlWRKY30 and SlWRKY81 that act positively in tomato response to *Ralstonia solanacearum* infection (RSI) [53], indicating the involvement of B-box-containing proteins in plant immunity. However, whether and how these B-box-containing proteins are involved in coordination of plant immunity and thermotolerance remain unclear.

Pepper (*Capsicum annuum*) is a vegetable of great agricultural importance [56], bacterial wilt caused by RSI and HTS are two important stresses in pepper production, in particular in tropical and subtropical regions [56, 57]. We previously found that CaWRKY40 acts positively not only in pepper immunity against RSI but also in defense response to HTS by targeting various immunity or thermotolerance-related genes via W-box in their promoter as well as by autoregulation at transcription level by the DWE, in its own promoter [58]. Upon RSI or HTS exposure, CaWRKY40 forms transcription cascades with TFs such as CaWRKY6, CaWRKY22, CabZIP23, and CabZIP63 [22, 23, 39, 59–61] as well as Ca^2+^ sensor CaCDPK15 [62], and is promoted by CaWRKY27b in pepper immunity against RSI and thermotolerance but by CaWRKY28 only in pepper immunity against RSI via physical interaction [41, 42]. In this way, CaWRKY40 forms positive feedback loops with WRKY TFs or other TFs to rapidly and efficiently activate immunity or thermotolerance upon perception of the stress. However, to function appropriately, some negative regulators might also be required. However, so far no negative regulators of CaWRKY40 have been identified. In the present study, a STH2 in pepper (CaSTH2) was found to act negatively in pepper immunity against RSI and in thermotolerance by repressing CaWRKY40’s interaction with CaWRKY27b/CaWRKY28.

## Results

### The expression assay of CaSTH2 upon RSI or HTS

A putative STH2 in pepper (*CaSTH2*) was originally found to interact probably with CaWRKY40 by pull-down combined with mass spectrum assay (Table S1, see online supplementary material), as STH2 was previously found to be transcriptionally modified by RSI in tomato and in pepper [53, 63], and CaWRKY40 acts positively in pepper immunity against RSI and thermotolerance [24]; consistently, it was also up-regulated by RSI and HTS (Fig. S1, see online supplementary material). According to our conjectures, *CaSTH2* may play a role in thermotolerance and pepper immunity against RSI. To verify the hypothesis, we measured the *CaSTH2* transcript level in pepper plant roots that had received an RS inoculation or not, and found that CaSTH2, which exhibits more than 95% sequence similarities to its orthologs in other plant species (Fig. S2A and B, see online supplementary material), was downregulated by RSI at 24 and 48 hpi (Fig. S2C, see online supplementary material). Given that pepper immunity against RSI and thermotolerance are closely related with each other in a way related to CaWRKY40 [24], to test whether *CaSTH2* is involved in the coordination of the two defense response, we tested the response of *CaSTH2* and found that *CaSTH2* was downregulated by HTS (Fig. S2C, see online supplementary material). In addition, by transient overexpression-based subcellular localization assay in *Nicotiana benthamiana* leaf epidermal cells, we found that CaSTH2 targets the plasma membrane and nucleus (Fig. S2D, see online supplementary material).

### The silencing of *CaSTH2* significantly enhanced pepper immunity against RSI and thermotolerance

**Figure 1:**
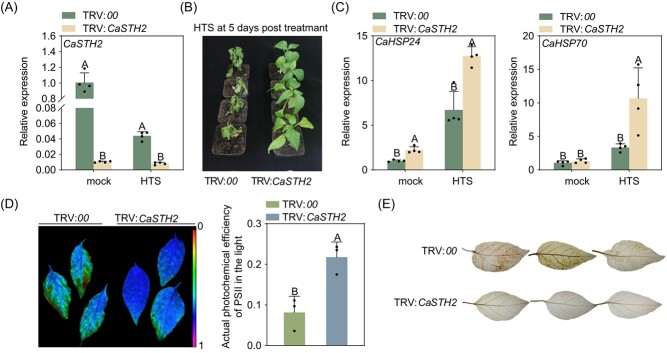
Impact of CaSTH2 silencing on pepper plants’ thermotolerance. (**A**) TRV:*CaSTH2* pepper plants exhibited much lower level of *CaSTH2* transcript with or without HTS treatment than TRV:*00* pepper plants. (**B**) TRV:*00* pepper plants were more sensitive to HTS than TRV:*CaSTH2* pepper plants. (**C**) Higher level of thermotolerance related *CaHSP24* and *CaHSP70* were found in the TRV:*CaSTH2* pepper plants than that in TRV:*00* pepper plants. (**D**) TRV:*CaSTH2* pepper plants exhibited higher levels of Fv/Fm and actual photochemical efficiency of PSII in the light, which are favorably associated with thermotolerance than the control. (**E**) TRV:*CaSTH2* pepper plants upon HTS accumulated much lower level of H_2_O_2_ displayed by DAB staining than TRV:*00* pepper plants. In **A**, **C**, and **D**, the data were normalized with *CaActin* serving as an internal reference. The results presented are the average ± SD of four replicates. On the bar graphs, distinct capital letters denote statistically significant differences (*P* < 0.01) between means based on Fisher’s least significant difference (LSD) test.

The transcriptional modification of *CaSTH2* by RSI and HTS implies its involvement in pepper immunity against RSI and in thermotolerance. To test this hypothesis, we assayed the function of *CaSTH2* by studying the effect of *CaSTH2* silencing on pepper immunity and thermotolerance using virus-induced gene silencing (VIGS) using two specific CDS regions for vector construction. RT-qPCR was used to verify that the silencing was successful, and found that the transcript level in TRV:*CaSTH2* plants was only 10–15% of that in the wild-type plants (Fig. 1A), and TRV:*CaSTH2* plants were more tolerant to HTS than the wild-type plants (Fig. 1B), as seen by higher level transcripts of thermotolerance-related *CaHSP24* and *CaHSP70* [64] (Fig. 1C), and higher level of Fv/Fm and photosystem II (PSII) photochemical efficiency in the light (фPSII) as well as lower level of H_2_O_2_ concentration displayed by DAB staining (Fig. 1D and E). All these data indicate that *CaSTH2* acts negatively in pepper tolerance to HTS. To confirm these results, we used another region in the 3' UTR of *CaSTH2* to do VIGS experiments (Fig. S3A and B, see online supplementary material), and the result consistently showed that *CaSTH2* silencing significantly increased pepper thermotolernace (Fig. S3C, see online supplementary material).

**Figure 2:**
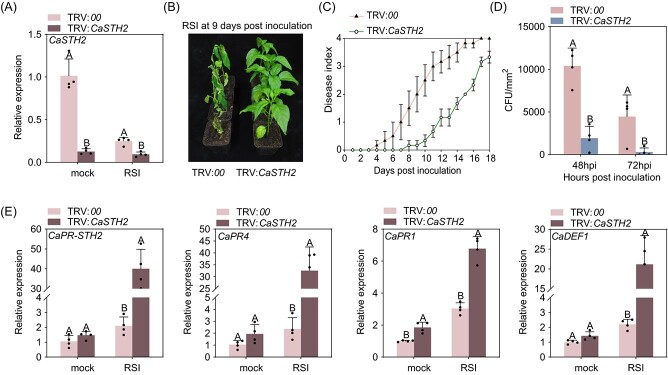
*CaSTH2* silencing enhanced pepper immunity against *Ralstonia solanacearum* infection. (**A**) TRV:*CaSTH2* pepper plants exhibited much lower level of *CaSTH2* transcript with or without *R. solanacearum* inoculation than TRV:*00* pepper plants by RT-qPCR. (**B**) TRV:*CaSTH2* pepper plants exhibited increased resistance to *R. solanacearum* inoculation than TRV:00 plants. (**C**) TRV:*CaSTH2* pepper plants inoculated by *R. solanacearum* exhibited much lower dynamic disease index(Table S3, see online supplementary material) than TRV:*00* plants from 2 to 18 dpi (days post inoculation), the disease index of 20 plants for each line and wild-type control plants were scored every day. (**D**) TRV:*CaSTH2* pepper plants inoculated by *R. solanacearum* supported much more bacterial growth than TRV:*00* plants. (**E**) TRV:*CaSTH2* pepper plants inoculated by *R. solanacearum* exhibited upregulated immunity related PR genes including *CaPR1, CaPR4* and *CaDEF1* than TRV:*00* plants. In **A**, **D**, and **E**, the data were normalized with *CaActin* serving as an internal reference. The results presented are the average ± SD of four replicates. On the bar graphs, distinct capital letters denote statistically significant differences (*P* < 0.01) between means based on Fisher’s least significant difference (LSD) test.

In addition, CaSTH2 was found to act negatively in pepper immunity against RSI, as the TRV:*CaSTH2* plants that exhibited significantly lower expression of *CaSTH2* upon RSI (Fig. 2A) also exhibited enhanced resistance to RSI at 9 days post inoculation (dpi) (Fig. 2B), displayed by lower dynamic disease index from 2 to 18 dpi (Fig. 2C), lower level of bacterial growth at 48 and 72 hpi (Fig. 2D), as well as upregulation of immunity-related *CaPR1*, *CaPR4*, and *CaDEF1* [23] in contrast to plants of the wild type (Fig. 2E). We also used another specific region in 5' UTR of CaSTH2 for VIGS vector contruction, and found that *CaSTH2* silencing line also reduced the pepper susceptibility to RSI (Fig. S3D).

### The transient expression of *CaSTH2* downregulated thermotolerance and immunity-related genes

To further confirm the result from VIGS, we assayed the effect of transient expression of *CaSTH2* on thermotolerance in pepper displayed by expression of thermotolerance related genes and Fv/Fm as well as фPSII, which are positively related to thermotolerance. The result showed that in CaSTH2-GFP transiently overexpressing pepper leaves, CaSTH2-GFP expressed successfully by western blotting using antibody of GFP (Fig. S4A, see online supplementary material), and this expression slightly reduced both HTS and RSI (Fig. S4B and C, see online supplementary material), and the transient overexpression of CaSTH2 reduced Fv/Fm and фPSII (Fig. S4D, see online supplementary material), and also downregulated thermotolerance-related genes including *CaHSP24*, *CaHSP70*, *CaHSFB2a* [64] (Fig. S4E, see online supplementary material). Additionally, the genes linked to immunity, such as *CaDEF1*, *CaPR1*, and *CaPR4* were all upregulated by RSI, but these upregulations were all repressed by *CaSTH2* transient overexpression (Fig. S4F, see online supplementary material). All these data support the result from VIGS that *CaSTH2* acts negatively in pepper thermotolerance and immunity against RSI.

### Overexpression of CaSTH2 significantly reduced thermotolerance and immunity of *N. benthamiana* against RSI

**Figure 3:**
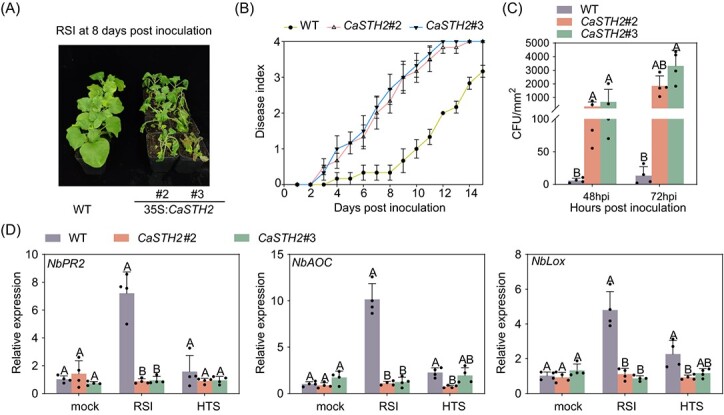
The overexpression of CaSTH2-GFP enhanced susceptibility of *Nicotiana benthamiana* plants to *Ralstonia solanacaerum*. (**A**) The CaSTH2-GFP overexpressing *N. benethamiana* plants exhibited enhanced susceptibilty to *R. solanacaerum* infection at 8 dpi. (**B**) The CaSTH2-GFP overexpressing *N. benethamiana* plants exhibited higher level of dynamic disease index from 2 dpi to 14 dpi, the disease index of 20 plants for each line and wild-type control plants were scored every day. (**C**) The CaSTH2-GFP overexpressing *N. benethamiana* plants supported higher level of bacterial growth than the wild type control plants at 48 and 72 hpi. (**D**) The CaSTH2-GFP overexpressing *N. benethamiana* plants upon *R. solanacaerum* inoculation exhibited higher level transcripts of immunity related genes including *NbPR2*, *NbAOC*, and *NbLox* at 48 hpi than the wild type plants. The data were normalized with *CaActin* serving as an internal reference. In **C** and **D**, the results presented are the average ± SD of four replicates. On the bar graphs, distinct capital letters denote statistically significant differences (*P* < 0.01) between means according to Fisher’s least significant difference (LSD) test.

We also functionally characterized CaSTH2 by ectopically expressing it in *N. benthamiana*. CaSTH2 was stably expressed in the two randomly selected T3 lines (Fig. S5, see online supplementary material). The plants of the two lines consistently demonstrated a higher vulnerability to RSI, displayed by higher levels of dynamic disease index from 2 to 14 dpi (Fig. 3B), higher levels of bacterial growth and lower levels of expression of the tested immunity-related genes including *NbPR2*, *NbAOC*, and *NbLox* [65] compared to the wild-type plants (Fig. 3C and D). In addition, the *CaSTH2* overexpressing *N. benthamiana* plants exhibited enhanced sensitiveness to HTS (Fig. S6A, see online supplementary material), lower level of Fv/Fm and фPSII and lower level of thermotolerance-related genes including *NbASC6*, *NbHSP18*, and *NbAPX* [66] (Fig. S6B–D). All the data from CaSTH2 ectopically expressing *N. benthamiana* plants support the conclusion that immunity against RSI and pepper thermotolerance are regulated adversely by CaSTH2.

### The confirmation of interaction between CaSTH2 and CaWRKY40

**Figure 4:**
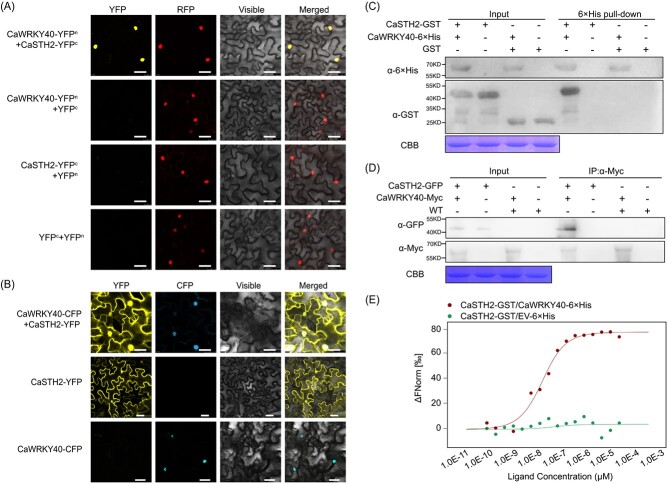
CaSTH2 interacted with CaWRKY40. (**A**) the data from BiFC using *Agrobacterium* cells bearing CaWRKY40-YFP^N^ + CaSTH2-YFP^C^ (using CaWRKY40-YFP^N^ + YFP^C^ and YFP^N^ + CaSTH2-YFP^C^ as negative controls) constructs were infiltrated into leaves of *Nicotiana benthamiana* leaves, and YFP signals were observed in epidermis cell of *N. benthamiana* leaves at 48 hpi. NbH2B (histone H2B)-RFP was used to indicate the nucleus. Bars =25 μm. (**B**) By agroinfiltration based transient overexpression in epidermis cell of *N. benthamiana* leaves, CaSTH2 and CaWRKY40 co-localized to the nuclei of epidermis cell of *N. benthamiana* leaves, Bars =25 μm. (**C**) CaSTH2 interacted with CaWRKY40 *in vitro* by pull-down assay using prokaryotically expressed CaSTH2-GST and CaWRKY40–6 × His, the CaSTH2 and its interacting protein were pulled down by Ni Smart beads and the existence of CaWRKY40 was found by immune blotting using antibody of GST. (**D**) CaSTH2 interacted with CaWRKY40 in CoIP assay using proteins isolated from CaWRKY40-Myc and CaSTH2-GFP co-transiently overexpressing leaves of pepper, and CaWRKY40 and its interacting proteins were immunoprecipitated with antibody of Myc and the presence of CaSTH2 in the isolated proteins was detected by immune blotting using antibody of GFP. (**E**) The interaction between CaSTH2 and CaWRKY40 was assessed using the MST assay, where CaSTH2-GST served as the target protein and CaWRKY40–6 × His was used as the ligand, with concentrations ranging from 1.0E^−10^ mM to 1.0E^−3^ mM. CaSTH2-GST/EV-6 × His or CaSTH2-GST/CaWRKY40–6 × His mixtures were put into Monolith NT.115 capillaries and subjected to measurements at room temperature with an LED excitation source with λ = 470 nm and 50% IR laser power.

To test and confirm the interaction between CaSTH2 and CaWRKY40, we first carried out BiFC to study the possible CaSTH2/CaWRKY40 interaction in planta. CaWRKY40-YFP^N^ and CaSTH2-YFP^C^ were produced by fusing the N- and C-terminal sections of YFP to CaWRKY40 and CaSTH2, respectively. In *N. benthamiana* leaves, the fusion proteins’ interaction was visible; the result showed that clear YFP signal was observed in the nuclei in epidermal cells of *N. benthamiana* leaves, indicating that CaSTH2 interacts with CaWRKY40 in the nuclei in planta (Fig. 4A). Consistently, CaSTH2 and CaWRKY40 were found to co-localize to the nuclei in epidermal cells of *N. benthamiana* leaves by agrobacterium infiltration-based subcellular localization assay (Fig. 4B). The interaction between CaSTH2 and CaWRKY40 was further confirmed by co-immunoprecipitation (CoIP) assay, Myc antibody was utilised to immunoprecipitate CaWRKY40 and potential interacting proteins from proteins extracted from CaSTH2-GFP and CaWRKY40-Myc co-transiently overexpressing *N. benthamiana* leaves, and an antibody of GFP was used to detect the presence of CaSTH2 by immunoblotting. The outcome revealed that CaSTH2 interacted with CaWRKY40 *in vivo* (Fig. 4D). With prokaryotic expressed CaSTH2-GST and CaWRKY40–6 × His, the CaSTH2/CaWRKY40 interaction was again validated *in vitro* by pull-down and micro thermophoresis (MST) assay. The results all showed that CaSTH2 interacts with CaWRKY40 *in vitro* (Fig. 4C and E). All the results collectively indicate that CaSTH2 interacts with CaWRKY40.

**Figure 5:**
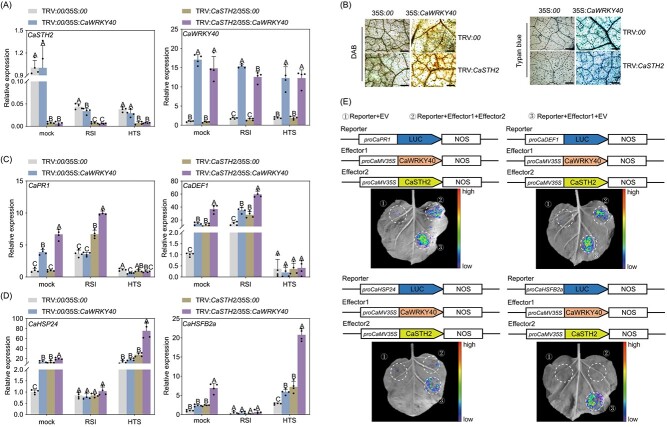
The activation of immunity or thermotolerance related genes by CaWRKY40 was repressed by CaSTH2. (**A**) The success of CaWRKY40 transient overexpression and *CaSTH2* silencing through virus-induced gene silencing in pepper plants by RT-qPCR assay. (**B**) Hypersensitive response-like cell death and H_2_O_2_ accumulation were observed by Trypan blue and 3,3′-diaminobenzidine (DAB) staining. Bars = 500 μm. CaWRKY40 was transient overexpressed in WT and *CaSTH2*-silenced pepper leaves, respectively, and silenced *CaSTH2* enhanced hypersensitive response. (**C**) The activation of immunity related genes including *CaPR1* and *CaDEF1* by CaWRKY40 was enhanced by *CaSTH2* silencing upon RSI at 48 hpi but not upon HTS by RT-qPCR. D. The activation of thermotolerance related *CaHSP24* and *CaHSP70* by CaWRKY40 was enhanced by *CaSTH2* silencing upon HTS at 48 hpt but not upon *Ralstonia solanacaerum* inoculation by RT-qPCR. E. The overexpression of *CaSTH2* significantly reduced the promoter activity of *CaPR1*, *CaDEF1*, *CaHSP24*, and *CaHSFB2a* that were enhanced by overexpression of *CaWRKY40*. *proCaPR1*, *proCaDEF1*, *proCaHSP24*, or *proCaHSFB2a*: luciferase and *35S: CaWRKY40* or *35S: CaWRKY40* + *35S: CaSTH2* were co-infiltrated into the leaves of *Nicotiana benthamiana.* At 48 hpi, the infiltrated leaves were harvested for luciferase imaging, and the relative activity of luciferase was measured. The data in A, C, and D were normalized using CaActin as the internal reference.The average ± SD of four duplicate results are shown. Based on Fisher’s least significant difference (LSD) test, discrete capital letters on the bar graphs denote statistically significant differences (*P* < 0.01) between means.

### *In vivo*, CaSTH2 inhibited CaWRKY40’s binding to target gene promoters that contained W-boxes, but not *in vitro*

The involvement of CaSTH2 and CaWRKY40 in both pepper thermotolerance and in immunity against RSI and their interaction imply that the role that CaWRKY40 plays might be modified by CaSTH2 via physical interaction. To test this speculation, we first performed electrophoretic mobility shift assay (EMSA) using biotin labeled W-box with promoter segments of *CaPR1*, *CaDEF1*, *CaHSP24*, and *CaHSFB2a*, which are targeted by CaWRKY40 [23, 64], and prokaryotic expressed CaSTH2–6 × His and CaWRKY40–6 × His. The result showed that CaWRKY40 bound to all of the tested promoter fragments, and the addition of excess CaSTH2 did not affect these bindings (Fig. S6A and B, see online supplementary material), indicating that CaSTH2 does not affect the targeting of CaWRKY40 to its target genes *in vitro*. We further assayed the effect of CaSTH2 interaction on targeting of CaWRKY40 to its targets in planta upon RSI/HTS by chromatin immunoprecipitation (ChIP)-qPCR using the chromatins isolated from CaWRKY40-Myc transiently overexpressing TRV: *CaSTH2* pepper plants upon RSI/HTS. The result showed that binding of CaWRKY40 to the promoters of its immunity or thermotolerance-related target genes were significantly increased by the silencing of CaSTH2 upon RSI/HTS (Fig. S7C and D, see online supplementary material), indicating that the targeting of CaWRKY40 to its target genes were repressed by CaSTH2 *in vivo* upon RSI/HTS.

In addition, the effect of *CaSTH2* silencing on regulation of immunity or thermotolerance-related target genes by *CaWRKY40* was also assayed using TRV:*CaSTH2* pepper plants (Fig. 5A). Transient overexpression of CaWRKY40 in leaves quickly triggered dense HR-like cell death in *CaSTH2*-silenced plants, which developed slowly in the control plants (Fig. 5B). The outcome showed that *CaWRKY40*’s transient overexpression increased the levels of immunity-related *CaPR1* and *CaDEF1* upon RSI but not upon HTS (Fig. 5C), while thermotolerance-related *CaHSP24* and *CaHSP70* were upregulated by transient overexpression of *CaWRKY40* upon HTS but not upon RSI (Fig. 5D), but these upregulations were all enhanced by *CaSTH2* silencing. Furthermore, we employed the LUC assay to examine the impact of CaSTH2 on the transcriptional regulation of the examined genes related to thermotolerance and PR. The result showed that all of the tested PR and thermotolerance-related genes were transcriptionally regulated by transient overexpression of CaWRKY40, but all of these upregulations were significantly repressed by the transient overexpression of CaSTH2 (Fig. 5E). These results collectively indicate that CaSTH2 represses CaWRKY40 in its targeting and activating the immunity/thermotolerance-related genes in a context-specific manner.

### The interaction between CaWRKY40 and CaWRKY27b/CaWRKY28 was repressed by CaSTH2

**Figure 6:**
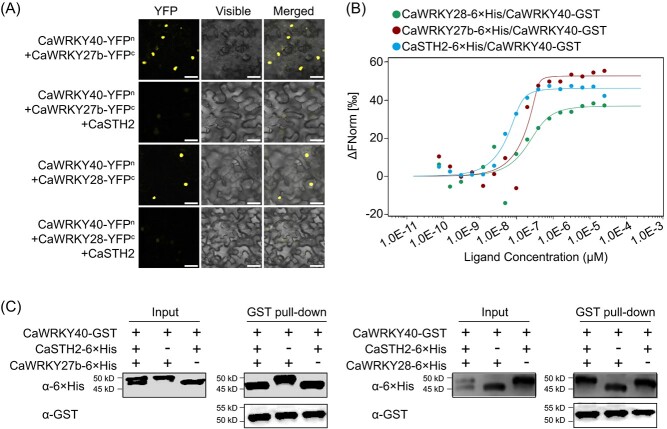
CaWRKY40-CaWRKY27b/CaWRKY28 interaction was blocked by CaSTH2. (**A**) CaWRKY40-CaWRKY27b/CaWRKY28 was blocked by co-transient overexpression of CaSTH2, CaWRKY40 and CaWRKY27b/CaWRKY28 by BiFC assay in leaves in *Nicotiana benthamiana* plants. (**B**) CaSTH2 exhibited a significantly higher binding affinity towards CaWRKY40 compared to both CaWRKY27b and CaWRKY28, as determined by the MST assay. In this case, the target proteins were considered as CaSTH2-/CaWRKY27b-/CaWRKY28–6 × His, while the ligand protein used was CaWRKY40-GST. C. The addition of CaSTH2 blocked the interaction between CaWRKY40-CaWRKY27b/CaWRKY28 in pull-down assays involving prokaryotically expressed CaWRKY27b/CaWRKY28–6 × His, CaSTH2–6 × His, and CaWRKY40-GST.

By previous studies, CaWRKY40 is promoted to activate PR genes such as *CaPR4*, *CaPR1*, *CaDEF1*, and *CaHSP24* by CaWRKY27b and CaWRKY28 [23, 58], and CaWRKY40 is repressed by CaSTH2 to activate the above-mentioned target genes not by repressing the direct targeting of these genes by CaWRKY40; we speculate that CaSTH2 might repress CaWRKY40 by blocking its interaction with CaWRKY27b or CaWRKY28. We used the BiFC assay to verify this assumption, and the results showed that the co-transient overexpression of CaSTH2 significantly reduced the CaWRKY40-CaWRKY27b/CaWRKY28 interaction (Fig. 6A), and the data from MST assay using the prokaryotic expressed proteins showed that CaSTH2 exhibited much higher levels of binding affinity to CaWRKY40 than CaWRKY27b and CaWRKY28 (Fig. 6B). In addition, the data from pull-down assays using the prokaryotic expressed proteins showed that that the addition of CaSTH2 blocked the binding between CaWRKY40 and CaWRKY27b/CaWRKY28 (Fig. 6C). All these data indicate that the interaction between CaWRKY40 and CaWRKY27b/CaWRKY28 might be repressed by CaSTH2, probably due to its priority in interacting with CaWRKY40.

## Discussion

By forming transcriptional cascades with other WRKY TFs or other TFs [22], CaWRKY40 plays a positive role in both thermotolerance and pepper immunity against RSI. Its function is further enhanced by CaWRKY27b in the context of the pepper immune response to RSI and HTS [41], and by CaWRKY28 exclusively in the context of pepper immunity against RSI [42] via physical interaction.

It can be speculated that negative regulators might also be required for CaWRKY40 to function appropriately, but so far, no such negative regulator has been reported. In the present study, we provide evidence that CaSTH2 acted as an interacting partner and a negative regulator of CaWRKY40 during pepper immune response to RSI and thermotolerance, with interaction between CaWRKY40 and CaWRKY27b/CaWRKY28 being blocked and thus targeting and activation of immunity or thermotolerance-related genes by CaWRKY40 being repressed.

### CaSTH2 has an adverse effect on thermotolerance and pepper immunity to RSI

Like results from previous studies in pepper and in tomato [67], STH2 was firstly found in a RNA-seq data set to be downregulated by RSI, which was further confirmed by the data from RT-qPCR that STH2 was significantly downregulated from 24 to 48 hpi (Fig. S2C, see online supplementary material). The data that STH2 silencing significantly enhanced pepper thermotolerance and immunity against RSI and the corresponding upregulation of thermotolerance or immunity related genes (Figs 1C and 2E) further confirmed the role of STH2 in pepper immunity against RSI, which was implied by its transcriptional downregulation (Figs 1C and 2E). By contrast, overexpression of CaSTH2 consistently reduced thermotolerance and immunity of pepper and *N. benthamiana* plants to RSI and consistent transcriptional modification of thermotolerance or immunity-related marker genes (Fig. 3E; Fig. S4D, see online supplementary material). So it can be speculated that in the absence of pathogen or HTS, the constitutive expressed STH2 might repress autoimmunity or thermotolerance to reduce fitness cost, when pepper plants are challenged with RSI or HTS, the repressed pepper immunity or thermotolerance might be derepressed by the downregulation of STH2. It is worth pointing out that the result that STH2 was downregulated by RSI is unlike the results from the previous studies that STH2 have been found to upregulated by RSI in tomato [53]. We speculate that the members in the STH2 family have functional divisions in pepper immune response to RSI; to confirm this speculation further investigation is needed. It is worth pointing out that our results indicate that *CaSTH2* silencing increased both pepper immunity and thermotolerance; however, by previous studies, plant immunity is generally repressed by HTS [68]. One explanation for this is that, as plant immunity and thermotolerance are two biological processes that are closely related, and a subset of regulatory proteins is shared in these two processes when plant is challenged by two stresses individually, and these shared players might act positively or negatively in the two processes [22, 39, 41, 42, 69], so the silencing of these proteins might result in increasing plant immunity and thermotolernace. However, upon the challenge of the two stresses simultaneously, plant immunity may be repressed by HTS.

### STH2 acts negatively in pepper thermotolerance and immunity against RSI partially through repressing the targeting of defense-related genes by CaWRKY40 via physical interaction

According to earlier research, CaWRKY40 activates PR or HSP genes and forms transcriptional cascades with CaWRKY6 [39], CaWRKY22 [59], CaWRKY30 [60], CabZIP23 [23], and CabZIP63 [22] to form positive feedback loops that act as protective buffers against RSI or thermotolerance. Additionally, CaWRKY40’s transcriptional activations of target genes related to immunity or thermotolerance are facilitated by CaWRKY27b [41] or CaWRKY28 [42]. In the present study, like CaWRKY27b and CaWRKY28, our data from BiFC, pull-down, CoIP, and MST assays all showed that CaSTH2 interacted with CaWRKY40 in the nucleus (Fig. 4), and the data from ChIP-qPCR showed that the enrichment of CaWRKY40 to the promoters of immunity or thermotolerance-related genes (Fig. S6C and D, see online supplementary material), and the transcript levels of these targeted genes were thus reduced by transient expression of STH2 (Fig. S4E and F, see online supplementary material), suggesting that the targeting and transcriptional activation of the target genes by CaWRKY40 were repressed by STH2 via physical interaction. Interestingly, this repression is not due to the direct repression of CaWRKY40 binding to the promoters of its target genes by CaSTH2 (Fig. S7A and B, see online supplementary material). Similarly, STH2 was previously found to act as a transcriptional regulator of HY5 to activate the chalcone isomerase gene in a way related to B-boxes in STH2 and G-box element in its target gene [46, 47, 70]. Thus, it can be speculated that STH2 might act as a transcriptional regulator generally in the crosstalk between plant photomorphogenic development and immunity, as photomorphogenesis and immunity can be regulated by shared regulatory factors such as Brassinosteroid (BR) [71], PUB3 [72], COP1 [47, 73, 74], calmodulin 7 [75], and HY5 [76], although this speculation needs to be confirmed in the future.

### CaSTH2 disables CaWRKY40 to activate thermotolerance or immunity-related genes by blocking the interaction between CaWRKY40 and CaWRKY27b/CaWRKY28

As CaSTH2 alone did not repress CaWRKY40 to bind the promoters of the tested immunity or thermotolerance-related genes *in vitro* (Fig. S7A and B, see online supplementary material), and addition of STH2 significantly repressed the interaction between CaWRKY40 and CaWRKY27b/CaWRKY28 (Fig. 6A and C), CaSTH2 exhibited higher binding affinity to CaWRKY40 than that of CaWRKY27b/CaWRKY28 (Fig. 6B), it can be concluded that CaSTH2 might prevent CaWRKY40 from activating its target genes related to immunity and thermotolerance by blocking its interaction with CaWRKY27b/CaWRKY28, as CaWRKY40 is promoted by these two proteins via physical interaction by our previous studies [41, 42]. This repression might be reduced by the downregulation of STH2 during the early peroid of RSI; however, at the later stage of RSI, the relatively high level of CaSTH2 might be required for preventing inappropriate activation of defense responses or for turning off the immune response once the invasion of pathogens has been deal with [77, 78].

## Conclusions

Collectively, our data indicate that CaSTH2 acts negatively in pepper immunity against RSI and thermotolerance by preventing CaWRKY40 from activating immunity and thermotolerance-related genes at least partially through blocking the interaction between CaWRKY40 and CaWRKY27b/CaWRKY28.

## Materials and methods

### Plant materials and growth conditions

The inbred pepper lines HN42 and *N. benthamiana* were planted in plastic pots with a soil mixture consisting of peat moss, perlite, and roseite, 1:1:1 v/v/v. The pots were then placed in a growth chamber with the following parameters: 28°C temperature, 70% relative humidity, 60–70 μmol photons m^−2^ s^−1^, and a photoperiod of 16 hours light followed by 8 hours dark.

### *R. solanacearum* inoculation and HTS treatment

*R. solanacearum* inoculation was carried out using the GMI1000 strain for root irrigation or for leaf injection. Roots were slightly mechanically damaged and then irrigated with 20 ml of *R. solanacearum* suspension diluted to 10^8^ cfu mL^−1^ (OD_600_ = 0.8) with deionised water. Leaf injection was performed by injecting 100 uL of *R. solanacearum* suspension liquid with a concentration of 10^4^ cfu ml^−1^ (OD_600_ = 0.4) with deionised water into the leaves and through the leaf veins using a disposable syringe. For the treatment of HTS, the plants are placed in a 42°C incubator for 48 hours of cultivation. The plants upon stress treatment are collected at specific time points for experimentation.

### Vectors construction

All vectors were constructed using Gateway cloning technology (Invitrogen, Carlsbad, CA, USA). Specific primer pairs for target genes were used to amplify full-length open reading frames (ORFs) or 3’UTR fragments (for virus-induced gene silencing) by PCR. After confirming the sequencing results, the amplicons were cloned into the entry vector pDONR-207 through BP reaction and subsequently transferred into destination vectors pEarleyGate-101/102/103 and pDEST-15/17 via LR reaction.

### Virus-induced gene silencing (VIGS) assay

Virus-induced gene silencing was performed following the method of Zhang *et al.* [52], vectors containing TRV1, TRV:*00*, TRV:*CaPDS* (positive control for PDS octadhydrolycopene dehydrogenase), and TRV:*CaSTH2* were transformed into GV3101 cells. The transformed GV3101 cells were collected by centrifugation and suspended in induction medium at an optical density of OD_600_ = 0.8. GV3101 cells carrying TRV:*CaSTH2*/TRV:*CaPDS*/TRV:*00* were mixed with cells carrying TRV1 at a ratio of 1:1 (v/v). Subsequently, each bacterial solution (100 μl) was infiltrated into cotyledons of pepper plants with three to four leaves. The infiltrated plants were then incubated in darkness at a temperature of 16°C and relative humidity of 70% for 56 hours. The efficiency of gene silencing for each vector was confirmed by qPCR using specific primer pairs when albino phenotypes appeared on leaves of TRV:*CaPDS* infiltrated pepper seedlings.

### Generation of transgenic *N. benthamiana* plants

The CaSTH2 overexpressing *N. benthamiana* plants for its gain-of-function assay were generated following the method of Regner *et al.* [79] using leaf discs as explants, for transformation with GV3101 cells containing 35S:CaSTH2-YFP, the T_0_ transgenic *N. benthamiana* were screened on 10 mM basta (glyphosate, Sigma-Aldrich, St. Louis, MO, USA) and then were validated with specific primer pair by PCR (Table S2, see online supplementary material). The acquired T_0_ plants were self-pollinated and their seeds were harvested for each plant individually. In a similar way, the seeds of T_2_ lines and T_3_ lines were acquired, the gain-of-function assay of *CaSTH2* was assayed using plants of homozygous T3 lines.

### Agrobacterium-mediated transient expression and subcellular localization assay

GV3101 cells containing specific expression vector such as 35S:CaSTH2-GFP (YFP) (using 35S:GFP (YFP) as a control) were suspended and diluted to OD_600_ = 0.6–0.8, and then injected into the leaves of pepper or *N. benthamiana* plants of 6–8 leaf stage using injection without a needle. For subcellular localization assay, the infiltrated leaves were harvested at 48 hpi and the GFP or YFP signal was observed by LEICA TCS SP8 confocal laser microscope.

### Bimolecular fluorescent complimentary (BIFC) assay

Using the freeze–thaw method, vectors of CaPR-STH2-YFPN and CaWRKY40-YFPC were transformed into Agrobacterium GV3101. The transformed GV3101 cells were amplified overnight and adjusted to OD_600_ = 0.6–0.8 using the induction solution (200 mM acetosyringone, 10 mM MES, 10 mM MgCl_2_, pH 5.4), and the GV3101 cells containing CaSTH2-YFP^N^ and CaWRKY40-YFP^C^ were mixed in a ratio of 1:1. The mixed GV3101 cells solution was placed on a shaker at 100 rpm at room temperature and shaken for 2–3 h. After mixing, *N. benthamiana* with suitable growth state was selected and the whole leaves were injected with the mixed bacterial solution. The tissue sections were observed under confocal microscope (TCS SP8, Leica, Wetzlar, Germany) 48 hours later.

### Co-inmunoprecipitation (CoIP) assay

CoIP was used in this study to verify CaSTH2-CaWRKY40 interaction *in vivo*, mix *Agrobacterium tumefaciens* containing CaSTH2-GFP and CaWRKY40-Myc in a 1:1 ratio, and then infect the leaves of 6–8 leaf age pepper plants; the total protein was extracted from the infiltrated leaves using the method described by Yang *et al.* [63]. The CaSTH2 and its possible interacting proteins were immunoprecipitated using monoclonal antibody-GFP magnetic beads (Sigma-Aldrich, St. Louis, MO, USA) following the instruction by Sigma-Aldrich, and existence of CaSTH2 and CaWRKY40 was verified by western blotting using the GFP antibody and the MYC-tagged antibody (Abcam, Cambridge, UK), respectively.

### Pull-down

Pull-down was used in this study to verify CaSTH2-CaWRKY40 interaction *in vitro* using the method of our previous studies [41, 80]. CaSTH2-GST and CaWRKY40–6 × His were prokaryotically expressed in and isolated from *Escherichia coli* (*E. coli*) strain BL21, which were mixed with BeaverBeads IDA-Nickel (Beaver Biosciences, Suzhou, Jiangsu, China) and incubated at 4°C for 3 hours, the beads collected, washed three times with wash buffer, and then elution buffer was used to elute the protein on the beads. In SDS-PAGE eluted proteins were separated and finally analysed by Western Bolt using GST and His antibodies (Sigma-Aldrich, St. Louis, MO, USA).

### Microscale thermophoresis (MST) assay

The MST technique was utilized to validate the interaction between CaSTH2 and CaWRKY40 *in vitro*, following a previously described [81] method. The fluorescent label was attached to prokaryotically expressed CaWRKY40–6 × His, while the non-fluorescent label was attached to CaSTH2-GST, as outlined by Huang *et al.* [80]. Various concentrations of CaSTH2-GST ranging from 1.0E–10 mM to 1.0E–3 mM were mixed with 20 mM of the labeled protein in an interaction buffer and incubated for 10 minutes. Subsequently, using an LED excitation source with λ = 470 nm, the samples were loaded into Monolith NT.115 Capillaries (Cat#MO-K002, NanoTemper Technologies, Munich, Germany) at room temperature and exposed to a 50% IR laser power. The data obtained were analysed using Nano Temper Analysis software version 1.2.20 to determine apparent Kd values [82, 83].

### Histochemical staining

DAB staining is used for hydrogen peroxide staining and the degree of hydrogen peroxide accumulation is indicated by the colour shade. Trypan blue staining was used to determine the degree of cell necrosis in the leaves, as described previously [39].

### Western blotting assay

Western blotting detects target proteins. The protein sample was mixed with the protein loading buffer at a volume of 4:1 and denatured at 95 degrees Celsius for 10 minutes. Due to the different size of the protein, it is separated in SDS-PAGE gel. Then, the protein was transferred from the gel to the PVDF membrane (Thermo Fisher Scientific, Waltham, MA, USA) at a constant current of 180 mA for 40 minutes using the semi-dry method (Bio-Rad), and the PVDF membrane was put into the blocking liquid and shaken at room temperature for 1 h. Subsequently, the membrane was incubated in 1:5000 diluted primary antibody (Abmart, Shanghai, China) at room temperature for 1 h. TBST buffer (TBS + 5% Tween 20) was then used to clean the membrane three times, each time for 5 minutes. Then the membrane was incubated in 1:20000 diluted secondary antibody at room temperature for 1 h, and the above operation was repeated three times with TBST buffer cleaning. Finally, the membrane was immersed in well-mixed ELC chemiluminescent solutions A and B (A: B = 1:1) for exactly one minute before capturing the image.

### RNA extraction and RT-qPCR assay

Total RNA extraction from pepper or *N. benthamiana* plants was performed using TRIzol reagent (Invitrogen, Canada). The extracted RNA concentration was standardized and cDNA templates were synthesized utilizing reverse transcriptase (TaKaRa, Tokyo, Japan). Relative transcript expression levels of target genes were detected using SYBR Premix Ex Taq on a Bio-Rad real-time polymerase chain reaction system (Bio-Rad Laboratories, Hercules, CA, USA). Specific primer pairs are listed in Table S2 (see online supplementary material). To normalize transcript expression levels during data analysis according to the Livak method [84], internal reference gene Ca*Actin* was employed.

### Chromatin immunoprecipitation (ChIP) assay

ChIP assay was used to study the enrichment of CaWRKKY40 in the promoters of its target genes following the method as previously described [85]. Briefly, GV3101 cells containing 35S:CaWRKY40-GFP or 35S:GFP were infiltrated into pepper leaves of TRV:CaSTH2 or TRV:00 plants upon RSI or HTS. Chromatin was extracted from leaves infected with Agrobacterium at 48 hpi and randomly broken into fragments of 300–500 bp using sonication. The fragment mixture was incubated with magnetic beads fused with GFP antibody (Sigma-Aldrich, St. Louis, MO, USA) for more than 2 hpi, then the DNA was collected from the magnetic beads and purified. Before confirmation by PCR and qPCR, the DNA fragments were further used in the ChIP-qPCR assay.

### Electrophoretic mobility shift assays

EMSA follows the previous method [58]. The probe was labeled with Cy5 dye (500 nM) and used for EMSA analysis together with CaWRKY40–6 × His and CaSTH2-GST fusion proteins. A total of 0.5 μg of fusion protein was mixed with the probe and incubated at room temperature for half an hour, then the above mixture in PAGE gel was separated and scanned with the Odyssey CLX imaging system (LI-COR).

### Luciferase assays

Promoter fragments of CaPR1, CaDEF1, CaHSFB2a, or CaHSP24 were inserted in front of the luciferase expression sequence in reporter vector pGreenII0800-LUC. CaWRKY40 and CaSTH2 were constructed into the expression vector pGreenII62-SK, and the above plasmids were transformed into Agrobacterium GV3101. *A. tumefaciens* containing *proCaPR1*-pGreenII0800-LUC, *proCaDEF1*-pGreenII0800-LUC, *proCaHSP24*-pGreenII0800-LUC or *proCaHSFB2a*-pGreenII0800-LUC, and pGreenII62-SK were mixed in 1:1 volume to co-infect *N. benthamiana* as negative control. *A. tumefaciens* containing *proCaPR1*-pGreenII0800-LUC, *proCaDEF1*-pGreenII0800-LUC, *proCaHSP24*-pGreenII0800-LUC or *proCaHSFB2a*-pGreenII0800-LUC and *CaWRKY40*-pGreenII62-SK were mixed in 1:1 volume to co-infect *N. benthamiana* as positive control. Then the positive control was mixed with the same volume of *CaSTH2*-pGreenII62-SK agrobacterium as the experimental group. Forty-eight hours after infection with *N. benthamiana*, fluorescein (1 mM) was applied to the leaves for CDD (charge coupled device, Nightshade LB985) imaging.

## Acknowledgements

We thank Mark D. Curtis for kindly providing the Gateway destination vectors, Dr S. P. Dinesh-Kumar of Yale University for the pTRV1 and pTRV2 vectors. This work was supported by grants from the National Natural Science Foundation of China (31902032, 31572136, 31372061) and Development Fund Project of Fujian Agriculture and Forestry University (CXZX2016158, CXZX2017548).

## Author contributions

S.H. and Xin.C. designed the research. M.W., Q.L., J.L., Y.S., and X.H. performed the experiments. S.Y., Y.Z., Y.H., R.W., and D.G. analysed the data. S.H. wrote the manuscript. All the authors of this paper read and approved the final manuscript.

## Data availability

The data that support the findings of this study are available in the Supporting Information.

## Conflict of interest statement

The authors declare that they have no conflicts of interest.

## Supplementary data

Supplementary data is available at *Horticulture Research* online.

## Supplementary Material

Web_Material_uhae066
