# Supplementary material for: CaSTH2 disables CaWRKY40 from activating pepper thermotolerance and immunity against Ralstonia solanacearum via physical interaction
Source: Hortic Res. 2024 Mar 2;11(5):uhae066. doi: 10.1093/hr/uhae066 (PMC11079491; doi:10.1093/hr/uhae066)
Supplement: Web_Material_uhae066 [file web_material_uhae066.zip › supplementary-information.docx]

**Table S2. Primers used in the present study.**

|  | **Gene** | **F primer** | **R primer** |
| --- | --- | --- | --- |
| **Primers used for *CaSTH2* and *CaWRKY* study** | ***CaSTH2*** | GGGGACAAGTTTGTACAAAAAAGCAGGCTTC ATGGGTATCAATACCTATGC | GGGGACCACTTTGTACAAGAAAGCTGGGTCTTAAGCGTAGACAGAAGGAT |
|  | ***CaSTH2-GFP*** | GGGGACAAGTTTGTACAAAAAAGCAGGCTTC ATGGGTATCAATACCTATGC | GGGGACCACTTTGTACAAGAAAGCTGGGTCAGCGTAGACAGAAGGAT |
|  | ***CaSTH2-VIGS1*** | GGGGACAAGTTTGTACAAAAAAGCAGGCTTCATGGGTATCAATACCTATGC | GGGGACCACTTTGTACAAGAAAGCTGGGTCTTTGACTTCATAAGTAATTG |
|  | ***CaSTH2-VIGS2*** | GGGGACAAGTTTGTACAAAAAAGCAGGCTTC TGAATGATGAAAAATGAATC | GGGGACCACTTTGTACAAGAAAGCTGGGTC AAATACTTTTCAAATGCTTA |
|  | ***CaWRKY40*** | CGGAATTCATGATGGAATTTACCAGTTTGGTTGA | CGGAATTCATGATGGAATTTACCAGTTTGGTTGA |
|  | ***CaWRKY40-GFP*** | CGGAATTCATGATGGAATTTACCAGTTTGGTTGA | CGGAATTCATGATGGAATTTACCAGTTTGGTTGA |
|  | ***CaWRKY27b*** | GGGGACAAGTTTGTACAAAAAAGCAGGCTTCATGGCTGAAAACCAAAACGA | GGGGACCACTTTGTACAAGAAAGCTGGGTC  TTAGTTGAACAGCAAAGTAGAGG |
|  | ***CaWRKY27b-GFP*** | GGGGACAAGTTTGTACAAAAAAGCAGGC  TTC ATGGCTGAAAACCAAAACGA | GGGGACCACTTTGTACAAGAAAGCTGGGTC  GTTGAACAGCAAAGTAGAGG |
|  | ***CaWRKY28*** | GGGGACAAGTTTGTACAAAAAAGCAGGCTT  CATGTCTGATAACCCTTTTTA | GGGGACCACTTTGTACAAGAAAGCTGGGTC  CTAGGATGGCTCTTGTTTCTGGA |
|  | ***CaWRKY28-GFP*** | GGGGACAAGTTTGTACAAAAAAGCAGGCTT  CATGTCTGATAACCCTTTTTA | GGGGACCACTTTGTACAAGAAAGCTGGGTC  CTAGGATGGCTCTTGTTTCTGGA |
| **Primers used for q-PCR analysis** | ***CaSTH2-*qPCR** | GGTGATCATGTTGTTACTGA | ACAGAAGGATTGGCGACGAG |
|  | ***CaWRKY40-*qPCR** | GGTGTGGCAGATGATAGTGC | CCAGGCACAACATCCAAGT |
|  | ***CaDEF1-*qPCR** | GTGAGGAAGAAGTTTGAAAGAAAGTAC | TGCACAGCACTATCATTGCATACAATTC |
|  | ***CaPR1-*qPCR** | GCCGTGAAGATGTGGGTCAATGA | TGAGTTACGCCAGACTACCTGAGTA |
|  | ***CaPR-STH2-*qPCR** | ACACACCCTAAAATC TCACTCCA | GCCTTAAACAAGAAGA AGAAAGAGCT |
|  | ***CaPR4-*qPCR** | CAACCCGCAGAACATCAACTGG | CCTCAAGCATCTACCGCAAGCA |
|  | ***CaHSP70-*qPCR** | TCTATTCTTCTGCACTTTGTGTACT | AGGACATAGAGATCTACATAGCTGT |
|  | ***CaHSP24-*qPCR** | GTTCGTCTAGCAGTTTGGTTCGGTTG | GTAATTTAACTAAACAGACTCTTACAACC |
|  | ***CaHSFB2a-*qPCR** | TTGTTGCTACTCCTTGCGCA | TGAAGAAGCTATGCGGCA |
|  | ***CaACTIN-*qPCR** | AGGGATGGGTCAAAAGGATGC | GAGACAACACCGCCTGAATAGC |
|  | ***NbLox*** **-qPCR** | ATTAACGTGGGCCATGCAGA | GCCGAAATTCAGCGAAGCAT |
|  | ***NbAOC*-qPCR** | ACAGCTTCTACTTCGGCGA | GGCAGATCCGGAATACCCTT |
|  | ***NbPR2*-qPCR** | TGATGCCCTTTTGGATTCTATG | AGTTCCTGCCCCGCTTT |
|  | ***NbASC6-*qPCR** | GCATTGTTATGAGTGGAGGGG | CAGATTCTAAGGCTTCTTTTGTGAC |
|  | ***NbHSP18-*qPCR** | AGAAACCCCAGATTCCCATA | GGCAGCCTAAACCTTCTCAT |
|  | ***NbAPX-*qPCR** | CGCTCCTCTTATGCTCCGTCTT | GGTGGCTCTGTCTTGTCCTCTC |
|  | ***NbPR-STH2-*qPCR** | TCACAACATCAGCTTCCCCA | CCCCCATCACCCTCAACAAT |
|  | ***NbDEF1-*qPCR** | GCCTTACCAAACCACCATGC | GCTGCAGCCAAAGTTTTTGC |
| **Primers used for ChIP-qPCR** | ***CaHSP24-*Tss** | TGAGTTTCTCGAATCCTTTTTCCC | TTCGCCGACTTAGCTTCACG |
|  | ***CaDEF1-*Tss** | TCTTTGCTCGTAATGATTTGTGACA | GGTGTTCTTGGCTTATAGTGGC |
|  | ***CaPR1-*Tss** | AGCTCCATCCCAAACCAACC | TGGTGTTGGGTCTGTGAGGC |
|  | ***CaHSFB2a-*Tss** | GAAGTTGCACATTTGACCATTGGGTTAG  CC | GGCTAACCCAATGGTCAAATGTGCAACTTC |
| **Primers used for LUC** | ***pCaHSP24-pGreenII 0800-LUC*** | GTCGACGGTATCGATAAGCTACAAACTAACTTACTTGTTGTAAC | CAGGAATTCGATATCAAGCTTTCTGCTACCAATCAAAGATTGCT |
|  | ***pCaDEF1-pGreenII 0800-LUC*** | GTCGACGGTATCGATAAGCTGTTATGATGCATAATATTAA | CAGGAATTCGATATCAAGCTTAATTTTCACAGACAGAGCT |
|  | ***pCaPR1-pGreenII 0800-LUC*** | GTCGACGGTATCGATAAGCTGGTATAGTGTATTGAATAAAACAA | CAGGAATTCGATATCAAGCTTTTGGGTTTTGGTGAGAATTAAGG |
|  | ***pCaHSFB2a-pGreenII 0800-LUC*** | GTCGACGGTATCGATAAGCTCTTTTTAATTTAATTGAATT | CAGGAATTCGATATCAAGCTATTTTCTTACCTGAAACACT |
|  | ***CaWRKY40-pGreenII 62-SK*** | CGATATCAAGCTATGGAATTTACCAGTTTGGT | CGGTATCGATAAGCTTTACCATCTGCCCGTCTGAT |

**Table S3. Grading standards for evaluation of disease resistance of pepper plants to *R. solanacearum* by root irrigation**

| **Score** | **Condition** |
| --- | --- |
| 0 | Pepper plant is normal and asymptomatic. |
| 1 | Plant has slight withering, the basal one or two leaves are withered, but the apical region of the plant is normal. |
| 2 | In addition to the top leaves, one or two leaves are withered, but the apical region of the plant is normal. |
| 3 | Two-thirds of the leaves of the pepper plant are withered, while the top of the plant is normal. |
| 4 | The whole plant is withered or dead. |

**Figures**


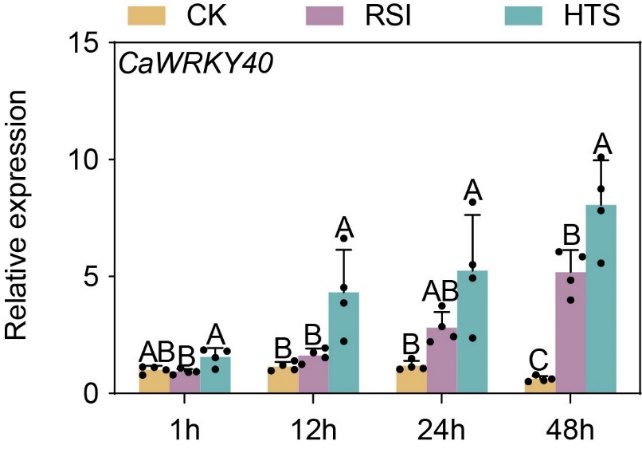


**Figure S1.** Transcriptional expression level of *CaWRKY40* in leaves of pepper plants upon *R.solanacaerum* inoculation at 1,12,24 and 48 hpi(hours post inoculation ) by RT-qPCR，data represent the mean ± SD of four replicates. *CaActin* was used as an internal control, different uppercase letters above the bars indicate significant differences between means (P< 0.01), as determined by Fisher’s protected least-significant-difference (LSD) test.


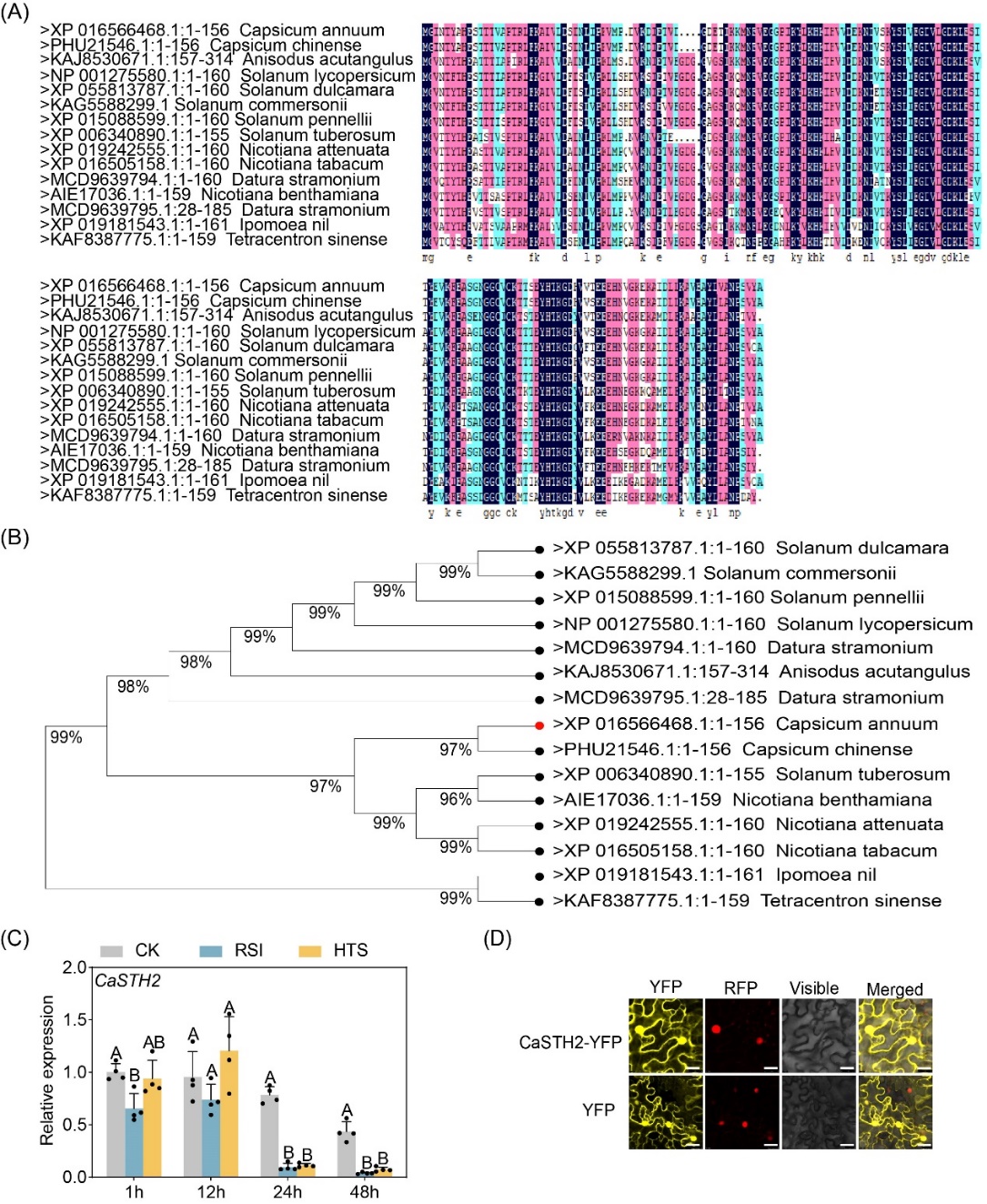


**Figure S2**. Deduced amino acid sequence of CaSTH2 and its expression assay. A. the multiple alignment of the deduced amino acid sequence of CaSTH2 with that of its orthologs in other plant species using DNAMAN8, where the black, red, blue and yellow shades indicated 100%, 75%-100%, 50%-75% and 33%-50% sequence similarity, respectively. B. phylogenetic tree of CaSTH2 deduced amino acid sequence with that of its orthologs in other Solanaceaes. C. transcriptional expression level of *CaSTH2* in leaves of pepper plants upon *R.solanacaerum* inoculation at 1,12,24 and 48 hpi(hours post inoculation ) by RT-qPCR，data represent the mean ± SD of four replicates. *CaActin* was used as an internal control, different uppercase letters above the bars indicate significant differences between means (P< 0.01), as determined by Fisher’s protected least-significant-difference (LSD) test. D. Subcellular localization assay of CaSTH2-YFP in epidermal cells of *Nicotiana benthamiana* leaves by agro-infiltration based transient overexpression, the YFP signal was observed by CLSM at 48 hpi(hours post infiltration) using YFP protein as negative control and RFP as a marker to indicate the nucleus, “visible” was the bright field cell field, “merged” is the first three superposition fields. Bars = 25 µm.


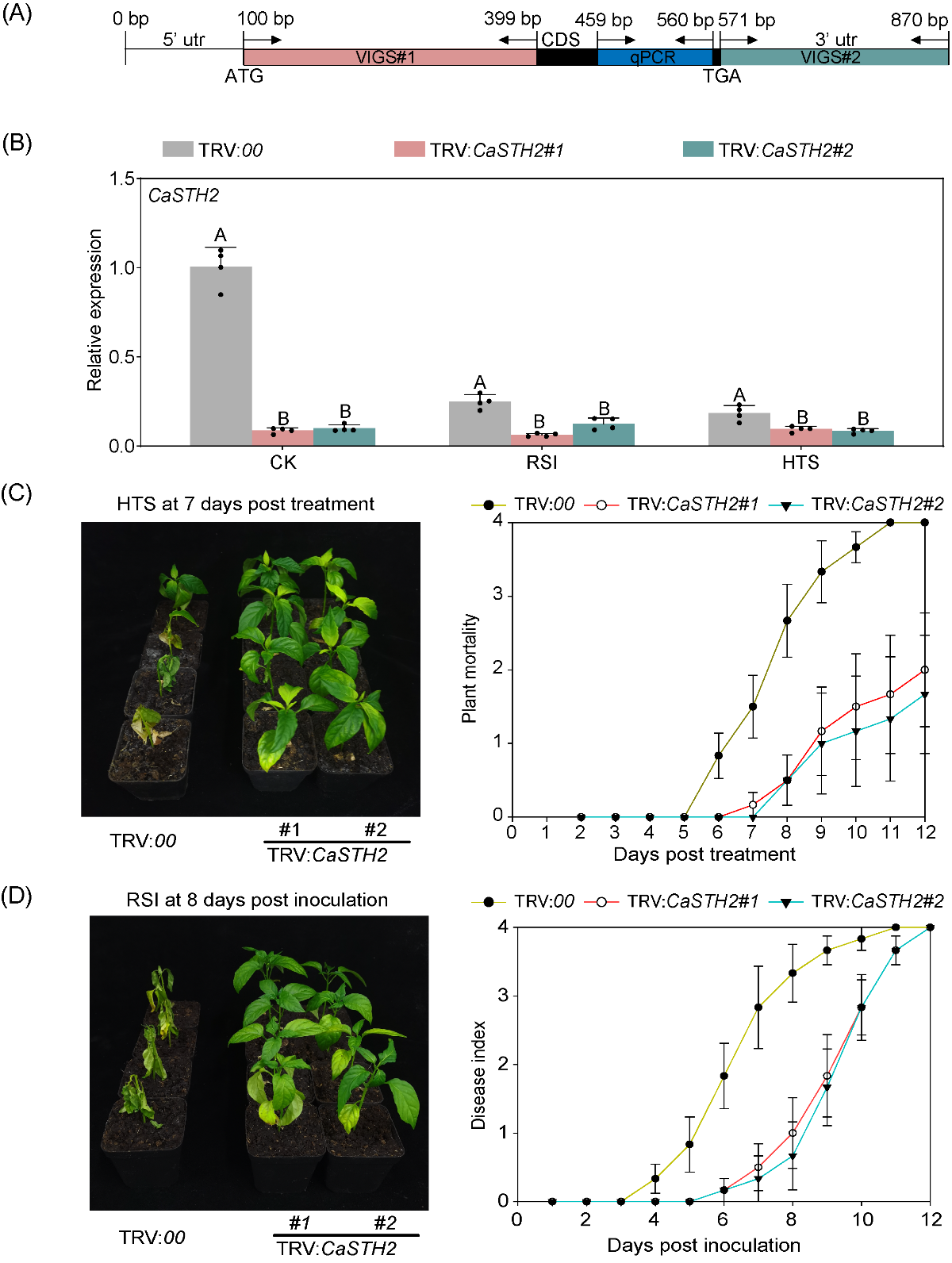


**Figure S3.** Effect of *CaSTH2* silencing on pepper thermotolernace and immunity. A. Two regions for vector contruction and one region for qPCR primer design in the CDS or 3'UTR of *CaSTH2*. B. TRV:*CaSTH2* pepper plants exhibited much lower level of *CaSTH2* transcript with or without HTS or RSI treatment than TRV:*00* pepper plants. C. TRV:*CaSTH2* pepper plants exhibited increased resistance to *R. solanacearum* inoculation than TRV:*00* plants. D. TRV:*00* pepper plants were more sensitive to HTS than TRV:*CaSTH2* pepper plants. In B, the data were normalized with CaActin serving as an internal reference. The results presented are the average ±SD of four replicates. Distinct capital letters on the bar graphs indicate statistically significant differences (P< 0.01) between means based on Fisher's least significant difference (LSD) test.


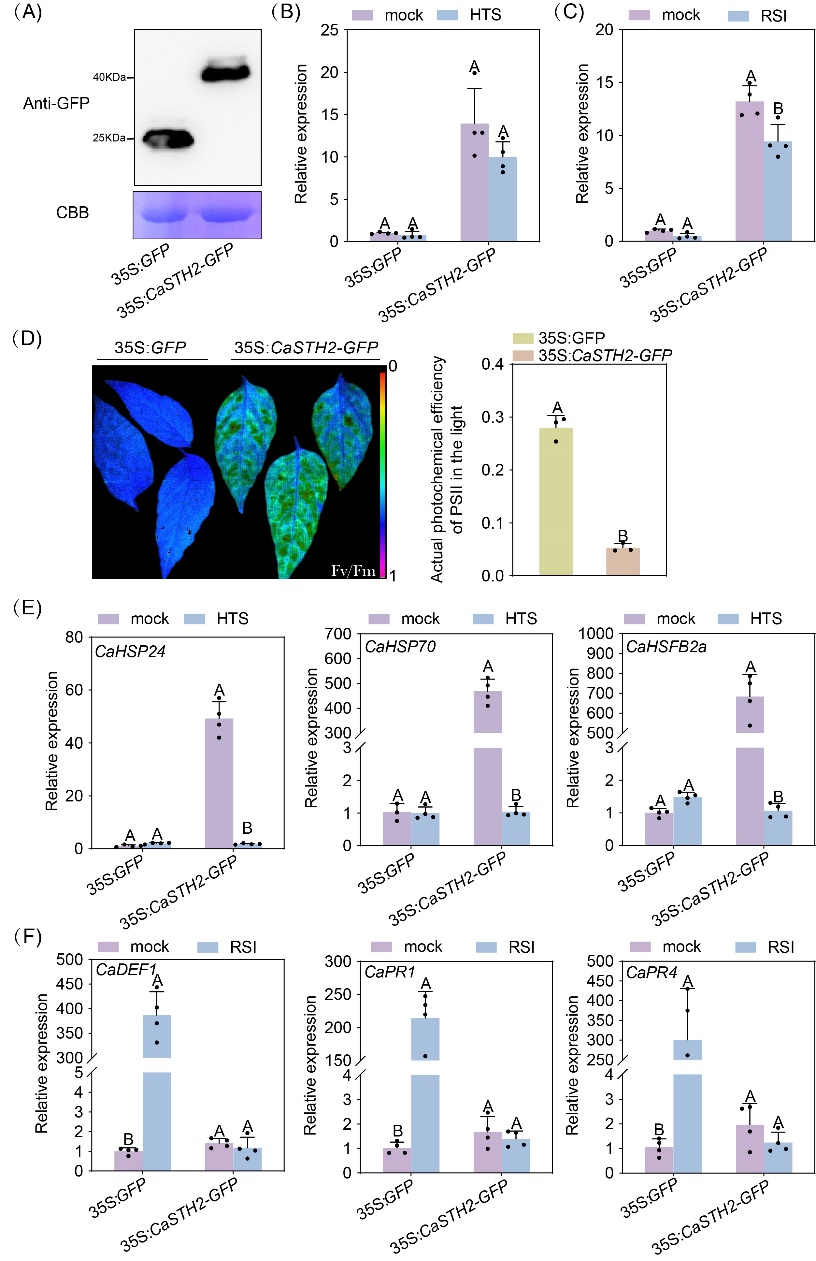


**Figure S4.** Transient expression of *CaSTH2* induced thermotolerance and immunity related genes upon HTS or *R.solanacaerum* inoculation. A. The success of CaSTH2-GFP expression in CaSTH2-GFP transiently overexpressing pepper leaves at 48 hpi(days post infiltration) by western blotting using antibody of GFP. B, and C. the *CaSTH2* transiently overexpressing pepper leaves exhibited higher level of transcript of *CaSTH2* with or without HTS treatment or *R.solanacaerum* inoculation at 48 hpt (i). D. the *CaSTH2* transiently overexpressing pepper leaves exhibited higher levels of Fv/Fm and actual photochemical efficiency of PSII in the light at 48 hpt than the mock treatment, data represent the mean ± SD of three replicates. E. the *CaSTH2* transiently overexpressing pepper leaves exhibited enhanced levels of thermotolerance related genes including *CaHSP24*, *CaHSP70* and *CaHSFB2a* upon HTS compared to the mock treatment. F. the *CaSTH2* transiently overexpressing pepper leaves exhibited enhanced levels of thermotolerance related genes including *CaPR1*, *CaPR4* and *CaDEF1* upon *R. solanacaerum* inoculation at 48 hpi compared to the mock treatment. In B, C, E and F, data represent the mean ± SD of four replicates. *CaActin* was used as an internal control, different uppercase letters above the bars indicate significant differences between means (P< 0.01), as determined by Fisher’s protected least-significant-difference (LSD) test.


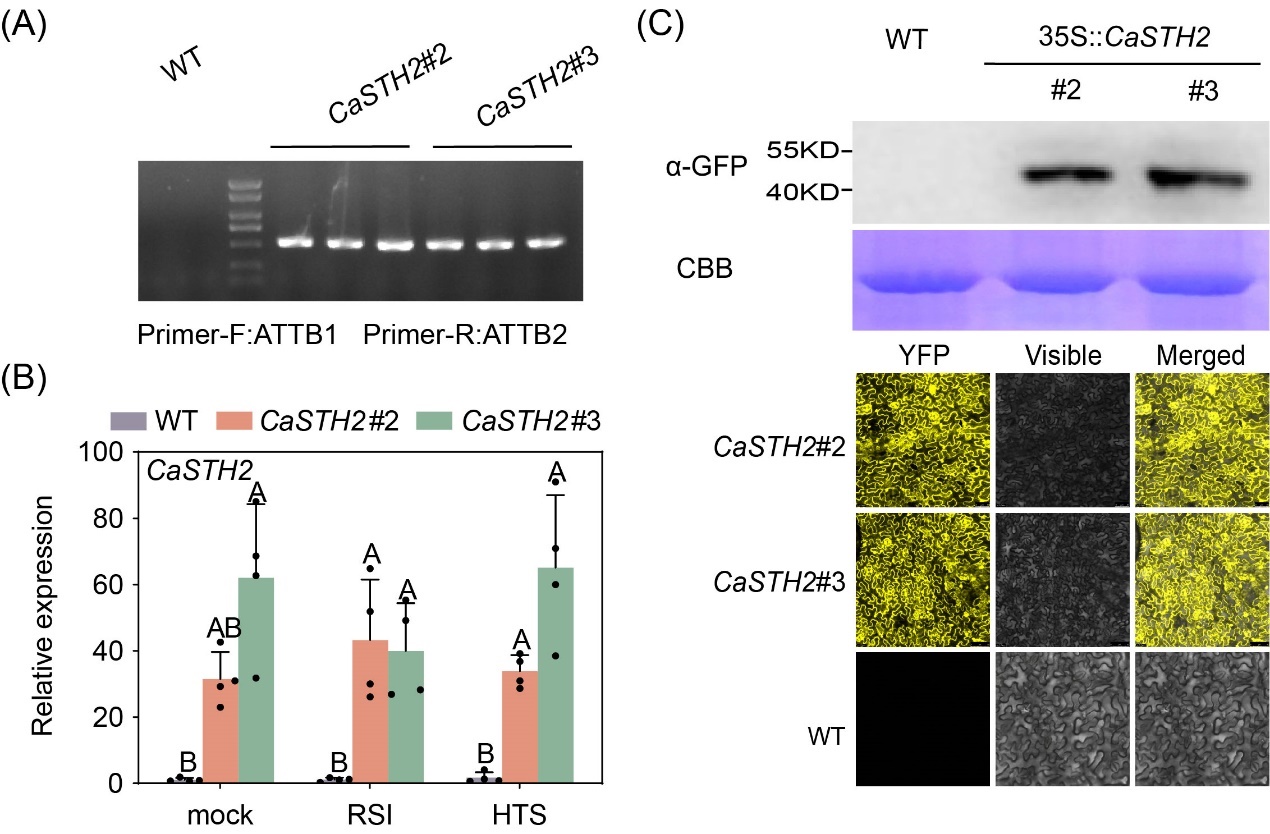


**Figure S5**.The confirmation of plants of two CaSTH2-overexpressing *Nicotiana benthamiana* lines. A. CaSTH2-GFP#2 and CaSTH2-GFP#3 were verified by PCR using specific primer pair of CaSTH2-GFP, B. CaSTH2-GFP#2 and CaSTH2-GFP#3 were verified by RT-qPCR using specific primer pair of CaSTH2-qPCR. C. CaSTH2-GFP#2 and CaSTH2-GFP#3 were verified by immune blotting using antibody of GFP and by detection of YFP signals in epidermal cells of the transgenic N.benthamiana leaves.


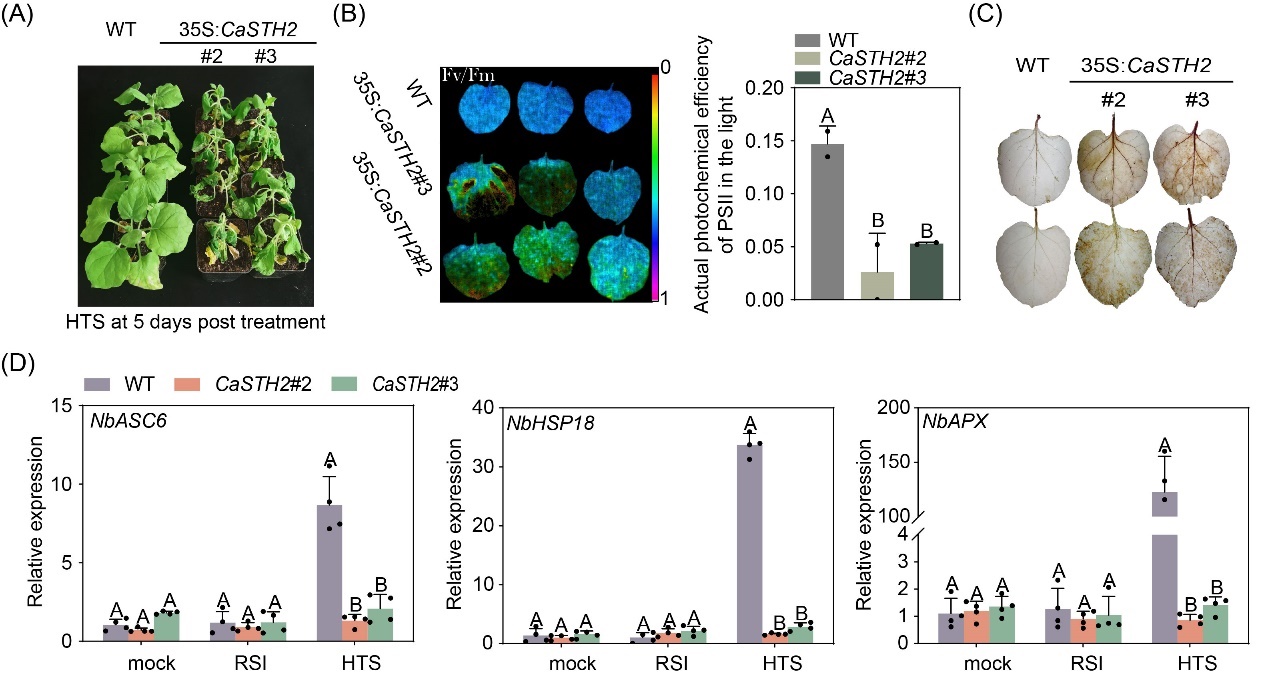


**Figure S6.** The overexpression of CaSTH2-GFP reduced thermotolerance of *N. benthamiana* plants. A. the CaSTH2-GFP overexpressing *N.benthamiana* plants exhibited reduced thermotolerance than the wild type plants. B. the CaSTH2-GFP overexpressing *N.benthamiana* plants exhibited reduced Fv/Fm and actual photochemical efficiency of PSII in the light upon HTS at 48 hpt compared to the wild type plants, data represent the mean ± SD of two replicates. C. the leaves of CaSTH2-GFP overexpressing N.benthamiana plants accumulated higher level of H_2_O_2_ displayed with darker DAB staining at 72 hpt than the wild type control. D. the CaSTH2-GFP overexpressing *N.benthamiana* plants exhibited lower level transcripts of thermotolerance related genes including *NbASC6*, *NbHSP18* and *NbAPX*. Data represent the mean ± SD of four replicates. *CaActin* was used as an internal control, different uppercase letters above the bars indicate significant differences between means (P< 0.01), as determined by Fisher’s protected least-significant-difference (LSD) test.


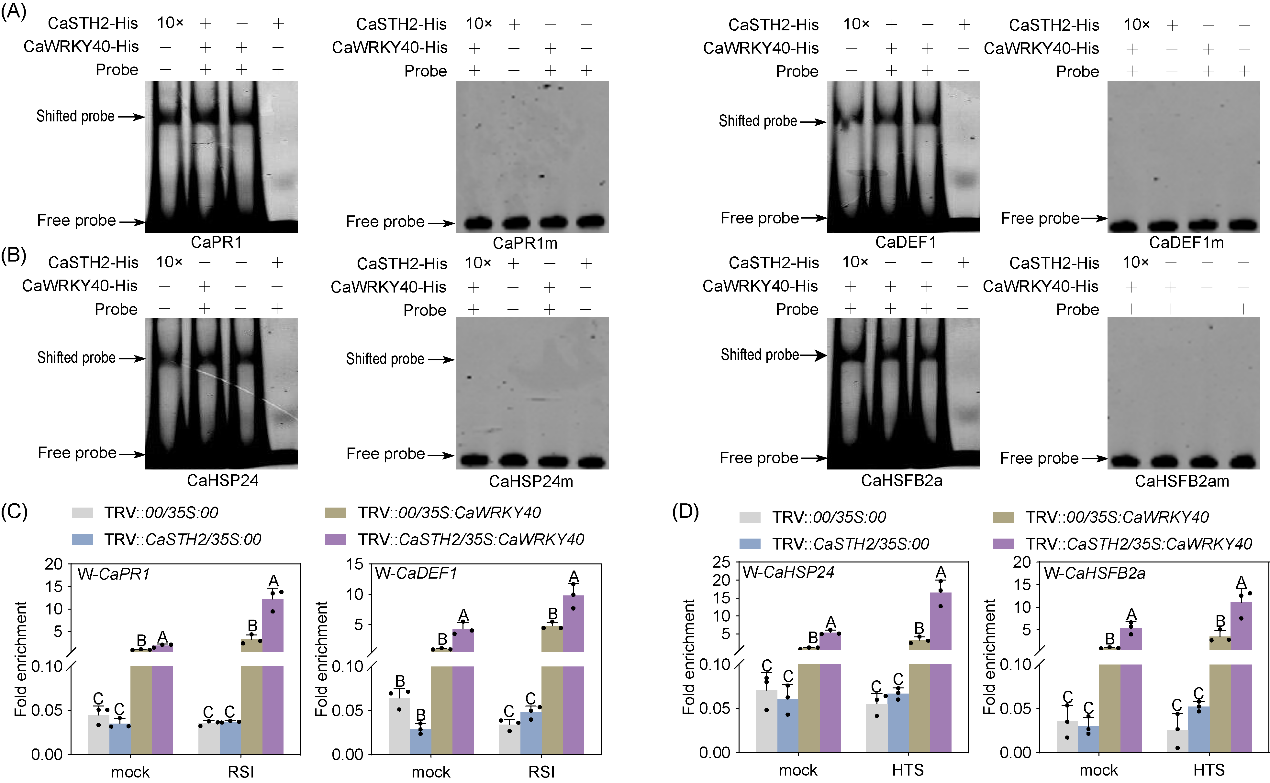


**Figure S7.** CaSTH2 did not affect the binding of CaWRKY40 and its immune-related or heat-resistant target gene promoters. A. CaSTH2 did not affect the binding of CaWRKY40 and its immune-related target gene *CaPR1* and *CaDEF1* promoters by Electrophoretic Mobility Shift Assay (EMSA) analysis using prokaryotic expressed CaSTH2-6×His and CaWRKY40-6×His and promoter fragments of immunity related genes including *CaPR1* and *CaDEF1* labelled with biotin. B. CaSTH2 did not affect the binding of CaWRKY40 and its thermotolerance-related target gene *CaHSP24* and *CaHSF2a* promoters by Electrophoretic Mobility Shift Assay (EMSA) analysis using prokaryotic expressed CaSTH2-6×His and CaWRKY40-6×His and promoter fragments of thermotolerance related genes including *CaHSP24* and *CaHSFB2a* labelled with biotin. C. the enrichment of CaWRKY40 to immunity related genes including *CaPR1* and *CaDEF1* were reduced by *CaSTH2* silencing upon *R.solanacaerum* inoculation by Chromatin-immunoprecipitation combined with qPCR (ChIP-qPCR). D. the enrichment of CaWRKY40 to thermotolerance related genes including *CaHSP24* and *CaHSFB2a* were reduced by *CaSTH2* silencing upon HTS by ChIP-qPCR. In C and D, data represent the mean ± SD of four replicates. Fold increases of immunoprecipitated DNA were calculated relative to the input DNA and the internal control *CaActin*, different uppercase letters above the bars indicate significant differences between means (P< 0.01), as determined by Fisher’s protected least-significant-difference (LSD) test.
